# Anaesthesiology and Ultrasound-Guided Injection of Botulinum Toxin in the Abdominal Wall: A State-of-the-Art and Technical Adaptation

**DOI:** 10.7759/cureus.79767

**Published:** 2025-02-27

**Authors:** Adriana Prezado Santos, Sara Matos, Lígia Reis

**Affiliations:** 1 Anaesthesiology, Unidade Local de Saúde Alentejo Central, Évora, PRT

**Keywords:** botulinum toxins, hernia, incisional hernia, interventional, pain management, ultrasonography, ventral

## Abstract

Ventral incisional hernia represents a prevalent postoperative complication, characterized by high morbidity and significant healthcare burden. Surgical management can be technically demanding, with a notable risk of recurrence and perioperative complications. This report describes a patient scheduled for hernioplasty with posterior component separation and bilateral transversus abdominis release. As an adjuvant technique, she was proposed botulinum toxin A (BTA) injection under ultrasound guidance into the three lateral abdominal muscle layers (transversus abdominis, internal oblique, and external oblique), to minimize tension and facilitate fascial medialization. Owing to their expertise in regional anatomy and proficiency in ultrasound-guided interventions, anaesthesiologists were consulted to perform the procedure. After four weeks, the size of the hernia decreased by 1.5 cm (width). On the day of surgery, combined anaesthesia was performed, with bilateral transversus abdominis plane block, followed by balanced general anaesthesia. The surgery lasted about five hours. There were no surgical complications, and the patient remained hemodynamically stable. The patient was transferred to the ICU under invasive mechanical ventilation. Ventilatory weaning was achieved after approximately 12 hours. Postoperative pain control was achieved through conventional analgesia, enhanced by the preoperative injection of BTA to alleviate muscle tension, avoiding neuraxial analgesia or intravenous opioids.

## Introduction

Ventral incisional hernias are a common complication associated with laparotomy, with an incidence of up to 30% [1]. The repair of these hernias can be challenging, especially in large-sized irreducible hernias and with chronic retraction of the muscles of the abdominal wall [2]. On average, 32% of patients have complications, and the recurrence rate is approximately 53% [3]. Component separation techniques are frequently employed to facilitate fascia medialization. However, these techniques have been associated with an increased risk of infection, dehiscence, and seroma formation [2]. Adjuvant techniques have been utilized, such as progressive pneumoperitoneum, the use of expanders, or the preoperative injection of botulinum toxin A (BTA), either alone or in combination [3,4].

BTA is a neurotoxin produced by the bacterium Clostridium botulinum that is known to inhibit the release of acetylcholine, which results in flaccid muscle paralysis with a maximal effect after four to six weeks [1]. The use of BTA in the lateral abdominal wall causes muscle relaxation of the lateral abdominal muscles, facilitating closure. This technique may decrease the need for mechanical separation, allowing the preservation of anatomical integrity and reducing the relapse and complication rates [3]. BTA can also reduce postoperative pain and the need for opioid analgesia, as it prevents postoperative muscle spasms [5] and inhibits the release of pain-modulating molecules from the pre-synaptic motor neuron’s terminal [1]. This procedure can be performed under ultrasound guidance to minimize risks and improve results, and anaesthesiologists’ support for such practice may become increasingly sought [6]. The adverse effects associated with this technique are minimal (pain at the injection site, abdominal distension sensation), with no reference to major complications, despite the theoretical risk of affecting ventilation. Nonetheless, the use of preoperative BTA is absolutely contraindicated in myasthenia gravis patients or in case of anaphylaxis to a component of the product used [6].

## Case presentation

The patient was a 70-year-old woman, American Society of Anesthesiologists Physical Status (ASA PS) II, with a diagnosis of complex ventral incisional hernia and a history of arterial hypertension, obesity (class II), and status post sigmoidectomy for volvulus with restoration of intestinal continuity one year ago. She also has a documented allergy to metamizole. The initial abdominal computed tomography (CT) revealed a ventral incisional hernia with 12 cm (width) x 19 cm (length) and intra-abdominal content procidence (intestinal loops). The Sabbagh and Carbonell indices were greater than 0.25 and 2.5, respectively. Hernioplasty with posterior component separation and bilateral transversus abdominis release (bilateral TAR) was proposed, with preoperative BTA injection four weeks prior to the surgery. Collaboration from the anaesthesiology team for the ultrasound-guided administration of BTA was requested, on an outpatient basis. The BTA was administered at six points (three points on each side of the abdomen), located between the midclavicular and anterior axillary lines (superior point - below costal margin at the midclavicular line; midpoint - equidistant from the superior and inferior points, at the anterior axillary line; inferior point - above the iliac crest, at the midclavicular line). For the procedure, we used a 12 MHz linear probe and a 100 mm echogenic single-shot peripheral nerve block needle. An in-plane needle approach was performed.

**Figure 1:**
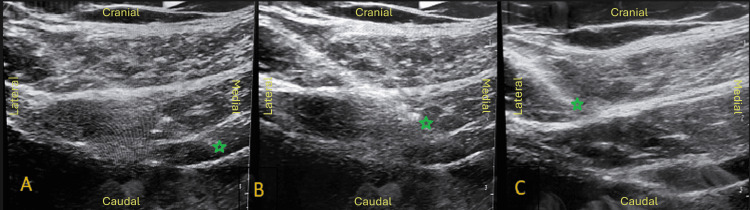
Sonoanatomy of the botulinum toxin A (BTA) infiltration applied to the three lateral abdominal muscle layers A: Transversus abdominis infiltration; B; Internal oblique infiltration; C: External oblique infiltration The green stars represent the tip of the echogenic single-shot peripheral nerve block needle.

At each point, even infiltration was performed in the three lateral abdominal muscle layers (Figure 1), from deep to superficial (i.e., first in the transversus abdominis, then in the internal oblique, and finally in the external oblique). In total, 300 U of Botox® (three 100 U vials) were administered. Each vial was diluted in 5 mL of normal saline (NaCl 0.9%), totaling 15 mL. Subsequently, 15 mL was diluted to make up 90 mL. At each point, 15 mL was injected (5 mL per muscle layer). The procedure took approximately 20 minutes, and the patient was under continual standard ASA monitoring for 45 minutes. There were no intercurrences, and the patient was discharged with the indication for hernia repair after four weeks.

**Figure 2:**
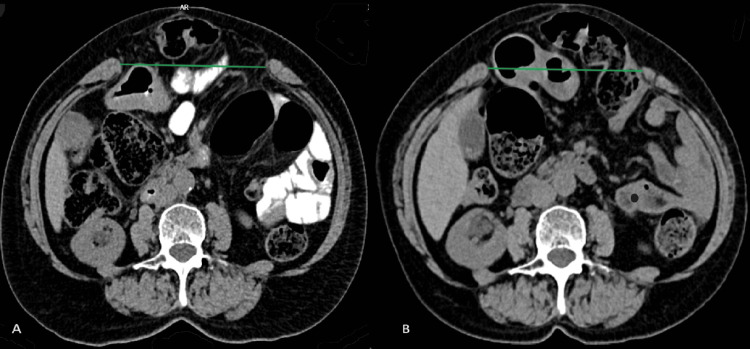
Transversal abdominal CT images showing incisional hernia before and four weeks after botulinum toxin A infiltration A: Before preoperative botulinum toxin A infiltration; B: Four weeks after preoperative botulinum toxin A infiltration The green lines represent the hernia width.

Two days prior to the surgery, a re-evaluation CT was performed (at about four weeks after BTA administration), revealing an incisional hernia with 10.5 cm (width) x 19 cm (length), presenting exteriorization of several intestinal loops and greater omentum’s adipose tissue (Figure 2), and an ICU postoperative admission was planned.

On the day of surgery, combined anaesthesia was performed, with bilateral transversus abdominis plane block with 0.2% ropivacaine (20 mL on each side), followed by balanced general anaesthesia. General anaesthesia was induced with 150 mg propofol, 150 mcg fentanyl, and 50 mg rocuronium, and sevoflurane was chosen for maintenance. Orotracheal intubation with videolaryngoscopy was used to secure the airway. Analgesia was supplemented with 30 mg ketorolac before surgical incision, 50 mcg fentanyl bolus, and 1 g acetaminophen. ASA standards monitoring were followed, and bispectral index and quantitative monitoring of neuromuscular blockade were also made. The surgery lasted around five hours, and the patient remained hemodynamically stable.

The patient was transferred to the ICU under invasive mechanical ventilation. Ventilatory weaning was achieved after approximately 12 hours, without incidents. At the ICU, the pain was controlled with 1 g acetaminophen t.i.d. and ketorolac 10 mg b.i.d. (visual analogue scale below 3). There were no complications, and the patient was discharged on day two of the ward. At the three-month follow-up, there was no clinical evidence of hernia relapse.

## Discussion

The use of BTA has become a safe and effective method of muscle relaxation, aiming to facilitate tension-free closure, prevent relapse, and mitigate the risk of complications, such as compartment syndrome and ventilatory restriction [3-6]. The criteria for preoperative BTA application include any ventral incisional hernia, in which primary fascial closure is unlikely to occur without the use of adjuvant techniques. The diameter of the hernia appears to be the main factor, followed by the loss of domain, defined as the protrusion of a significant portion of intra-abdominal content (percentages of 15-20% have been reported) [6]. In this case, it has been appropriately considered and utilized.

An optimized protocol for BTA administration has not yet been defined, with currently used protocols varying in terms of dose, number and location of administration, and preoperative timing. Usually, 500 U of Dysport® or 200-300 U of Botox® is injected in three to five locations on each side, on the three muscles of the lateral abdominal wall, at about four weeks before surgery [3-7]. The number and location of the applications that maximize muscle paralysis depend on the concentration of the BTA solution utilized. Most studies report the use of Botox® at 2-5 U/mL [6]. In this case, the concentration used was 3.3 (3) U/mL, an intermediate concentration, on three points per side, attaining satisfactory results in terms of hernia width reduction. The total Botox® dose was 300 U, with no adverse effects observed, such as dyspnea or a subjective weakness in coughing and sneezing. Therefore, despite the risk of the paralysis of these muscles negatively affecting the mechanics of breathing [1], an increase in pulmonary complications has not been observed, suggesting that this is a safe and effective dose. Nonetheless, as a disadvantage, despite not requiring significant physical and human resources or a significant amount of time, Botox® is a costly product.

There is only one case of iatrogenic botulism following ultrasound-guided BTA injection into the abdominal wall musculature. Five days after the procedure (Dysport®, 500 U, three to five injections per side, 50 U per injection), the patient presented to the emergency department with dysarthria, muscle weakness, constipation, and dyspnea, which rapidly progressed to respiratory failure, requiring invasive mechanical ventilation [8]. Ultrasonography presents several advantages, including ease of access, absence of radiation exposure, favorable cost-benefit, and moderately high precision by enabling real-time visualization of BTA dispersion and its anatomical relationship with adjacent vasculature. Although it reduces the likelihood of systemic absorption, it does not completely eliminate the risk. In obese patients, who are more predisposed to complex ventral hernias, the ultrasound technique may be significantly more laborious and require greater skill in probe-needle handling. It may even be necessary to use a convex probe and a lengthier needle. Real-time CT fluoroscopy can be an alternative in these circumstances [6]. The imaging approach chosen depends on resource availability and operator preference.

An approach to evaluate the efficacy of BTA use has not been standardized [5]. Several factors may be considered in combination, namely, pre- and post-administration imaging findings (specifically, the decrease of the defect and muscle thickness), intraoperative ease of achieving medial closure, intra- and postoperative complications, and the recurrence rate [3-5]. In this case, there was a 1.5 cm reduction in the defect’s width, there were no intra- or postoperative intercurrences, and, to date, there is no evidence of relapse. Regarding the imaging findings, it would have been relevant to have access to the variation in muscle thickness, which should decrease as a result of muscle fiber relaxation. The timing of evaluation by abdominal CT has not been established either [5]. A decision was made to perform it after four weeks, as it has been established that the average peak effect occurs at four to six weeks, and performing additional CT studies would result in unnecessary radiation exposure [1].

Concerning postoperative pain management, there was no need for neuraxial analgesia or intravenous opioid analgesia, often necessary in these procedures, particularly those carried out in this hospital center, without the use of adjuvant techniques. It highlights the benefits of intramuscular administration of BTA in achieving improved surgical and anaesthetic outcomes, enabling better pain control without the need for neuraxial anaesthesia.

Due to the prolonged surgical duration and the inherent risk of abdominal compartment syndrome associated with the procedure [3], along with the patient's history of obesity (which increases the risk of difficult airway management and ventilatory restriction), it was decided to postpone extubation. This approach aimed to facilitate a controlled and safer ventilatory weaning process, minimizing potential perioperative respiratory complications. However, the goal remained early weaning, which was successfully achieved in the ICU. With greater familiarity among the anaesthesia, surgery, and intensive care teams, extubation in the operating room may be a viable option in the future.

## Conclusions

The preoperative administration of BTA is an adjuvant technique that can reduce the need for more invasive surgical procedures, improving outcomes and reducing morbidity. It is a low-risk and quick procedure (on average, takes less than 30 minutes), associated with minimal discomfort, and can be performed on an outpatient basis, in the ward. Anaesthesiologists are familiar with the ultrasound technique and the anatomy of the abdominal wall, as they perform ultrasound-guided analgesic blocks that involve the interfascial planes of these muscles.

We report the successful preoperative administration of BTA four weeks before bilateral TAR, performed by anaesthesiologists, with a reduction in the hernia's dimensions, no periprocedural complications, and likely obviating the need for neuraxial anaesthesia. This case highlights the crucial role of anaesthesiologists' expertise in ultrasound-guided procedures, not only in perioperative locoregional analgesia but also in assisting preoperative optimization strategies. Furthermore, we emphasize the importance of multidisciplinary alignment and close collaboration between general surgery and anaesthesiology. As this is not a routine technique in anaesthesiology practice, anaesthesiologists must remain up to date with current literature on protocols, surgical and analgesic benefits, and potential associated risks.
